# Unveiling the Molecular Architecture of HBV Spherical Subviral Particles: Structure, Symmetry, and Lipid Dynamics

**DOI:** 10.3390/v17010048

**Published:** 2024-12-31

**Authors:** Sonal Garg, Alyssa Ochetto, Jianming Hu, Joseph Che-Yen Wang

**Affiliations:** Department of Microbiology and Immunology, Penn State College of Medicine, Hershey, PA 17033, USA; sjg6389@psu.edu (S.G.); amo5910@psu.edu (A.O.); juh13@psu.edu (J.H.)

**Keywords:** hepatitis B virus, subviral particle, surface antigen, cryo-EM, lipid bilayer, particle assembly, antigenicity

## Abstract

Since the discovery of the Australia antigen, now known as the hepatitis B surface antigen (HBsAg), significant research has been conducted to elucidate its physical, chemical, structural, and functional properties. Subviral particles (SVPs) containing HBsAg are highly immunogenic, non-infectious entities that have not only revolutionized vaccine development but also provided critical insights into HBV immune evasion and viral assembly. Recent advances in cryo-electron microscopy (cryo-EM) have uncovered the heterogeneity and dynamic nature of spherical HBV SVPs, emphasizing the essential role of lipid–protein interactions in maintaining particle stability. In this review, recent progress in understanding the molecular architecture of HBV SVPs is consolidated, focusing on their symmetry, lipid organization, and disassembly–reassembly dynamics. High-resolution structural models reveal unique lipid arrangements that stabilize hydrophobic residues, preserve antigenicity, and contribute to SVP functionality. These findings highlight the significance of hydrophobic interactions and lipid–protein dynamics in HBV SVP assembly and stability, offering valuable perspectives for optimizing SVP-based vaccine platforms and therapeutic strategies.

## 1. Introduction

Hepatitis B virus (HBV) infection remains a critical global health concern, capable of causing chronic disease and life-threatening complications. According to the World Health Organization (WHO), approximately 254 million people worldwide are living with chronic HBV infection as of the latest estimates [1]. Nearly half of these infections occur among individuals aged 30–54, a demographic whose healthcare needs impose significant economic strain. Recent data from 187 countries emphasize the magnitude of this challenge; in 2022, hepatitis B was responsible for an estimated 1.1 million deaths globally, averaging approximately 2956 deaths per day, while 1.2 million new infections were reported, despite extensive vaccination programs. While preventative vaccines have proven highly effective, current antiviral therapies achieve a functional cure in only 5–10% of cases, leaving the majority of patients reliant on lifelong treatment [2].

**Figure 1:**
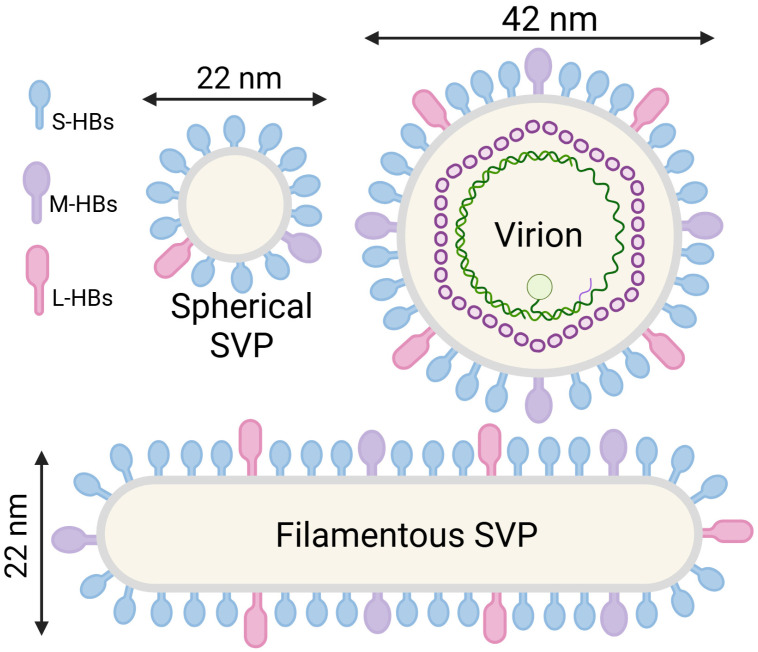
Schematic presentation of HBV virion and SVPs. A schematic illustration of the infectious HBV virion, spherical SVP, and filamentous SVP. The infectious HBV virion, 42 nm in diameter, contains an icosahedral nucleocapsid housing the partially double-stranded DNA genome and a reverse transcriptase. This nucleocapsid is surrounded by a lipid envelope embedded with the surface proteins, S-HBs, M-HBs, and L-HBs. In contrast, SVPs comprise a host-derived lipid membrane and surface proteins. Spherical SVPs primarily consist of S-HBs, with minimal amounts of M-HBs and trace levels of L-HBs, whereas filamentous SVPs and virions have higher proportions of M-HBs and L-HBs. The approximate S-HBs:M-HBs:L-HBs ratios are 4:1:1 in filamentous SVPs and 5:2:3 in virions. Created with BioRender.

HBV is a small, enveloped DNA virus in the *Hepadnaviridae* family that primarily infects hepatocytes. Its genome, a relaxed circular, partially double-stranded DNA, is enclosed in an icosahedral capsid and surrounded by a host-derived lipid envelope containing the hepatitis B surface protein (HBs) (Figure 1, upper right) [3], which is also referred to as hepatitis B surface antigen (HBsAg). When discussed collectively, particularly in the context of their assembly into particles or their antigenic properties, they are referred to as HBsAg. This distinction is maintained throughout the manuscript to clarify the context. Originally identified as the “Australia antigen”, the HBsAg plays a crucial role in viral entry and immune recognition [4,5]. Remarkably, HBsAg is found not only on infectious virions but also in vast quantities on non-infectious subviral particles (SVPs), which appear as spheres and filaments under transmission electron microscopy (TEM) (Figure 1) [6,7]. Produced at concentrations 1000–100,000 times higher than virions, SVPs act to saturate the immune system, diverting antibodies away from infectious particles, a property that has been instrumental in vaccine development [8].

The first HBV vaccine, approved in 1981, was based on HBV SVPs purified from patient plasma. To ensure safety, inactivation processes for early plasma-derived vaccines went beyond boiling and included chemical treatments such as cesium chloride purification and formalin treatment, as used in the Hevac B Pasteur vaccine [9]. These methods preserved immunogenicity, enabling the production of protective antibodies [10,11]. A second-generation recombinant vaccine, introduced in 1986, employed yeast cells to express cloned HBV surface antigens, producing particles that mimicked natural HBsAg. This breakthrough provided robust protection against HBV infection and remains a cornerstone of global vaccination programs [12]. Research into the structure and function of HBsAg and SVPs continues to advance our understanding of HBV infection mechanisms and immune interactions.

## 2. The Molecular Biology of HBsAg

**Figure 2:**
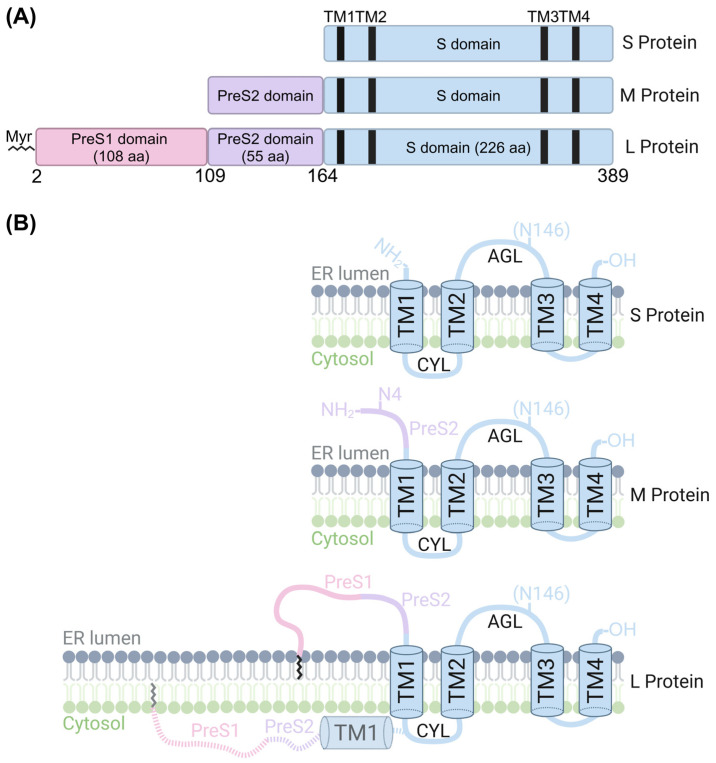
Schematic presentation of surface protein topology. (**A**) Sequence composition of the HBV surface proteins (HBs). The S domain, shared by all three proteins (S, M, and L), contains four transmembrane helices (TM1–TM4). The M protein includes an additional PreS2 domain (55 amino acids), while the L protein contains both PreS1 (108 amino acids) and PreS2 domains. The N-terminal glycine (G2) of the L protein undergoes N-myristylation (zigzag line), a post-translational modification essential for interaction with the NTCP receptor. (**B**) Proposed topologies of the HBs proteins. All three proteins share a common topology within the S domain, which features a cytosolic loop (CYL) and an antigenic loop (AGL). Half of the N146 residues in the S domain are N-glycosylated, as indicated by the parentheses (N146). In M-HBs, the additional N4 residue in the PreS2 domain is glycosylated, but this glycosylation is absent in L-HBs [17]. The diagrams illustrate the orientation of the proteins across the ER membrane, with the PreS1 domain of L-HBs exhibiting dual topology [18,19]. Created with BioRender.

HBsAg consists of three surface proteins, small (S), middle (M), and large (L), that collectively form the viral envelope. These proteins are encoded by a single open reading frame (ORF) in the HBV genome, which contains several in-frame ATG codons. Translation initiation from these codons produces the L protein (preS1, preS2, and S domains), M protein (preS2 and S domains), and S protein (S domain alone) (Figure 2A). The L protein is translated from the 2.4-kb subgenomic RNA, while the more abundant 2.1-kb RNA directs the production of the M and S proteins, ensuring a sufficient production of these essential components [13]. Despite differences in their N-terminal sequence lengths, all three proteins share a conserved C-terminal S domain. This domain contains the immunodominant “a”-determinant loop, located between residues 99 and 169 of the S domain, which mediates binding to the low-affinity receptor—heparan sulfate proteoglycans (HSPGs). The “a”-determinant is also the primary target of neutralizing antibodies, making it critical for immune recognition and the basis for HBsAg vaccines [14].

In spherical SVPs, the S protein is the most abundant, with smaller amounts of the M protein and minimal incorporation of the L protein. In contrast, filamentous SVPs and virions have higher proportions of M and L proteins, reflecting their structural and functional roles [15]. The relative distribution of HBsAg (S:M:L) differs significantly between particles, with virions having a ratio of 3:2:5 and filamentous SVPs exhibiting a ratio of 1:1:4 [16].

### 2.1. The Small Surface Protein (S-HBs or S Protein)

S-HBs consists of the conserved S domain, which includes four transmembrane helices and spans 226 amino acids (Figure 2B, S protein). This domain anchors the envelope proteins to the membrane and is essential for structural integrity [14]. Studies using in vitro translation systems have demonstrated that the S protein is an integral membrane protein, with its “a”-determinant loop partially translocated into the lumen of the endoplasmic reticulum (ER) [20,21]. As the primary envelope protein, S-HBs is sufficient to drive SVP production when expressed alone. It is the predominant surface protein in both spherical and filamentous SVPs, as well as in virions, far surpassing the levels of L and M proteins.

### 2.2. The Middle Surface Protein (M-HBs or M Protein)

The M-HBs includes the S domain along with a 55-amino-acid pre-S2 domain extension at the N-terminus [22]. During translation, the pre-S2 domain is translocated to the ER lumen (Figure 2B, M protein), while the S domain retains the same transmembrane topology as S-HBs [23]. The pre-S2 region contributes to the antigenicity and functionality of M-HBs in virion assembly and immune interactions [16,24]. Like S-HBs, M-HBs can be expressed and secreted independently, although the morphology of the particles produced solely by M-HBs remains unclear [25,26]. However, the M-HBs is not required for HBV replication, virion morphogenesis, or infectivity [16,27].

### 2.3. The Large Surface Protein (L-HBs or L Protein)

The L-HBs contains both the S and pre-S2 domains, with an additional pre-S1 domain at the N-terminus (Figure 2, L protein), varying from 108 to 119 amino acids in length depending on the HBV genotype [28]. The pre-S1 domain mediates binding to sodium taurocholate cotransporting polypeptide (NTCP), the primary HBV receptor on hepatocytes [29]. N-terminal myristylation, a lipidation modification, is vital for L-HBs interaction with the NTCP hepatocyte receptor [30]. This post-translation modification consists of the covalent attachment of a myristoyl (14-carbon saturated fatty acid) to the N-terminal glycine residue to enhance the protein stability and hydrophobicity of L-HBs. A unique aspect of N-terminal myristylation of HBV includes facilitating L-HBs dual topology within the membrane. This L-HBs dual topology consists of the pre-S domains orienting cytoplasmically before post-translationally translocating to an approximately equal distribution between cytoplasmic and ER luminal orientations [31,32].

## 3. Morphogenesis of HBV SVPs

HBsAg morphogenesis is a highly regulated process within hepatocytes, yielding infectious HBV virions and non-infectious SVPs. During translation, the S, M, and L proteins integrate into the ER membrane. Nucleocapsid-containing virions bud into the ER-Golgi intermediate compartment (ERGIC), enveloped by HBsAg, before trafficking to multivesicular bodies (MVBs) for exocytosis via the ESCRT pathway [33,34,35]. Conversely, SVPs derived from excess HBsAg bud directly into the ERGIC. SVPs undergo glycosylation in the Golgi apparatus before exosomal release from MVBs [36]. HBsAg is indispensable for HBV infectivity and immune evasion. In virions, it mediates nucleocapsid–envelope interactions during virion formation and is essential for infectivity by mediating host cell binding and entry [15]. In SVPs, it determines the particle structure, forming spherical or filamentous shapes depending on the relative abundances of S, M, and L proteins. These decoy SVPs sequester neutralizing antibodies, facilitating chronic infection.

## 4. Mystery of Lipid Organization in HBV SVPs

### 4.1. Lipid Composition of SVPs

The HBV SVPs isolated from human serum are stable lipoprotein complexes resistant to chemical, physical, and enzymatic treatments [37]. Their lipid composition has been reported differently depending on the focus of the study. One study exclusively quantified specific phospholipid classes, identifying a composition of phosphatidylcholine (PC, 65%), sphingomyelin (SM, 30%), and lysophosphatidylcholine (LPC, 5%), with no detectable phosphatidylethanolamine (PE) or phosphatidylserine (PS) [38]. Glycolipids, such as GSL-1, enhance immunogenicity by influencing antigenic determinants. In contrast, a broader lipid profiling study reported that patient-derived SVPs consistently exhibit lipid profiles enriched with PC (67%), cholesterol ester (14%), cholesterol (15%), and triglycerides (3%) [39]. Cell culture-derived SVPs display distinct lipid profiles. Hepatoma cell-derived SVPs contained over 90% phospholipids, with 80% PC and ether-linked lipids, reflecting altered metabolism in infected cells [40]. Yeast-derived SVPs lack SM but contain unsaturated fatty acids, while mammalian Chinese hamster ovarian cells (CHO)-derived particles contain PC, PE, phosphatidylinositol (PI), LPC, and SM. Mouse fibroblast-derived SVPs showed 90% PC and minor cholesterol (3%), contrasting with cholesterol-rich host membranes [41,42].

Reconstitution experiments demonstrated the critical role of acidic phospholipids, such as PS, in restoring antigenic activity and stabilizing epitopes near lipid–protein interfaces. Two antigenic determinant groups have been identified, the lipid-dependent and protein conformation-dependent groups, highlighting host-origin influences on lipid composition [41,42]. While lipids stabilize the structure of SVPs, antigenicity remains protein-dependent [37,43], as delipidation reduces helical content and diminishes antigenic activity [44].

### 4.2. Comparison with Lipoproteins

Lipoproteins, such as low-density lipoproteins (LDLs) and high-density lipoproteins (HDLs), are protein–lipid complexes essential for lipid transport through the bloodstream. These particles feature a phospholipid monolayer with PC and other phospholipids organized on the surface, providing a fluid and dynamic interface [45]. The enzymatic activity demonstrates that the lipids in LDL are readily accessible and dynamic, consistent with a flexible monolayer structure [46]. Despite extensive lipid remodeling, lipoprotein integrity is maintained by stabilizing protein–protein interactions. Given the similarity in lipid composition, early studies hypothesized HBV SVPs might resemble lipoproteins. Both LDL and HBV SVPs are PC-rich, but analyses revealed that the lipids in SVPs include triglycerides, cholesterol esters, free cholesterol, and phospholipids, resembling the lipid profile of HDL [39]. However, HBV SVPs differ fundamentally in structural dependence: while LDL disassembles upon lipid removal, SVPs maintain particle integrity, indicating reliance on protein–protein interactions rather than lipid monolayer dynamics [43].

HBV SVPs also feature a distinct lipid organization. Unlike the lipid monolayer of lipoproteins, SVPs have a non-bilayer arrangement where lipids are immobilized by strong lipid–protein interactions [41]. Enzymatic studies show that while phospholipases can access some surface lipids, the phospholipids in SVPs do not undergo the typical exchange seen in lipoproteins, further confirming their immobilization [41,42]. This rigidity suggests that lipids in SVPs serve a structural role, diverging from the transport-oriented functionality of lipoproteins. Fluorescence spectroscopy studies further highlight differences between LDLs and HBV SVP, as LDLs have a phospholipid monolayer surrounding a hydrophobic core that is more polar and fluidic than HBV SVPs [47]. Additionally, the S-HBs extends deeply into the lipid core, contrasting with the surface-bound apolipoprotein in LDLs. These findings highlight fundamental differences in lipid–protein organization, with HBV SVPs representing a specialized adaptation distinct from lipoproteins.

### 4.3. The Lipid Organization Debate: Monolayer or Bilayer?

The unique lipid–protein organization of HBV SVPs challenges the conventional monolayer paradigm observed in lipoproteins. HBsAg particles contain approximately 75% protein and 25% lipid, with lipids interspersed within protein aggregates, resulting in a discontinuous bilayer (or lipid patches) rather than a uniform lipid sheet [48]. Studies with hydrophobic (DPH) and polar (TMA-DPH) probes confirm rigid lipid immobilization [48], supported by electron spin resonance data [41]. These findings suggest that HBV SVPs adopt a lipid organization distinct from canonical monolayers, with lipids serving a stabilizing rather than a dynamic role. This structural adaptation features the functional divergence of HBV SVPs from other viral and lipoprotein complexes.

## 5. Symmetry and Structure of Spherical SVPs

The structural heterogeneity of spherical SVPs was first documented in the late 1960s and early 1970s when electron microscopy revealed particle sizes ranging from 18 to 22 nm in diameter in SVPs isolated from human patients [7,49,50,51]. These particles appeared roughly spherical, with a mosaic-like surface composed of subunits. Negative-staining TEM indicated an average particle diameter of 18.3 nm, with subunits measuring 7.5 nm and spaced 10 nm apart [52]. However, more recent high-resolution cryo-electron microscopy (cryo-EM) studies have refined these measurements and provided more accurate models of SVP structure, as discussed later. Freeze-fracture microscopy observed slightly larger particles (~19.6 nm) featuring ring-shaped surface subunits ranging from 6 to 8 nm in diameter [52]. While twofold and threefold symmetries were apparent, there was no conclusive evidence of fivefold symmetry.

X-ray scattering studies provided further insights into the SVP organization. In low-density solvents, SVPs appeared non-spherical, while in high-density solvents, theoretical predictions aligned with experimental data, suggesting a permeable particle shell. Dense, globular protein subunits were on the surface, while lipids appeared homogeneously distributed [52]. Variations in size and shape were attributed to differences in lipid–protein composition, incomplete assembly, or particle degradation. Yeast-derived SVPs displayed distinct structural features, including a larger diameter (27.5 nm) and a thicker cortex (~7–8 nm), compared to the human-derived particles, which measured 21.2 nm in diameter and had a 6.7 nm cortex [53].

Atomic force microscopy (AFM) provided additional structural details of recombinant SVPs derived from yeast. These particles were predominantly spherical, with diameters ranging from 20 to 27 nm [54]. Approximately 75 spike-like protrusions were observed per particle, spaced 5.8 nm apart, though no clear symmetry was evident. These protrusions, confirmed to be proteinaceous, altered their appearance upon treatment with disulfide bond-disrupting agents, such as dithiothreitol (DTT).

**Figure 3:**
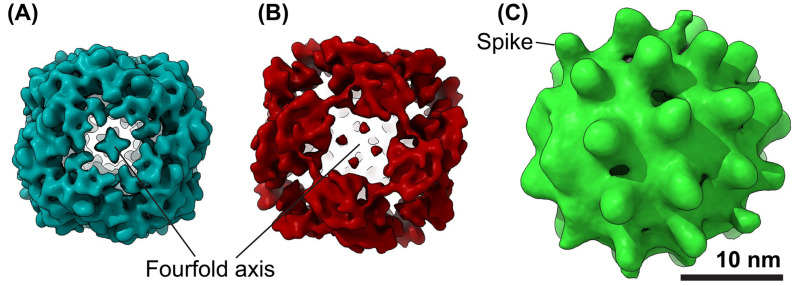
Cryo-EM 3D reconstruction of HBV SVPs. Cryo-EM 3D reconstructions of (**A**) the small SVP (EMDB code: 1158) and (**B**) the large SVP (EMDB code: 1159) purified from transgenic mice, resolved to 12 Å, from Gilbert et al. [55]. (**C**) Cryo-EM 3D reconstruction of the B map (EMDB code: 6953) from Cao et al., purified from an HBV carrier at ~30 Å [57]. The protruding spike appears localized to one side of the particle. While the surface lattice and morphology of the protruding spike in this map closely resemble those reported in other publications [60], the smaller SVP described by Cao et al. [57] is significantly larger than the SVPs reported by Gilbert et al. [55]. Scale bar, 10 nm.

Advances in cryo-EM have provided critical insights into the symmetry and structure of HBV SVPs. Analysis of SVPs isolated from transgenic mice revealed two particle sizes, measuring 19.6 nm and 21.2 nm in diameter, exhibiting octahedral symmetry characterized by twofold, threefold, and fourfold rotational axes [55]. The tightly packed S-HBs subunits form the twofold axes, whereas large open spaces form the fourfold axes (Figure 3A,B). Density variations between smaller and larger particles were most evident at the threefold locations. Despite these advances, the heterogeneous size distribution and unconventional lipid organization of SVPs pose significant challenges for achieving high-resolution structures. Subunit interactions modeled using analogous proteins, such as bacteriorhodopsin, propose that the twofold interactions involve HBsAg dimers. Consequently, SVPs likely comprise of 48 protein subunits [55].

Further cryo-EM analysis of yeast-derived recombinant HBsAg SVPs revealed predominantly spherical particles with slightly amorphous boundaries, measuring approximately 20–22 nm in diameter. A 3D reconstruction resolved to 15 Å with octahedral symmetry identified 24 “knuckle”-like protrusions on the surface. Each protrusion comprised tetrameric S-HBs subunits, representing nearly twice the number of subunits proposed in earlier models [55]. The following two distinct lipid regions were observed: an ordered outer monolayer (~2.5 nm thick) and a more variable, amorphous inner lipid layer. The S-protein protrusions, partially embedded within the lipid monolayer, formed a tightly packed framework stabilized by transmembrane helix–helix and lipid–protein interactions. These protrusions house critical antigenic epitopes, including cysteine-rich loops essential for immunogenicity [56].

The cryo-EM structure of spherical SVPs derived from HBV carriers, resolved at ~30 Å, revealed symmetrically arranged spike-like protrusions on the surface (Figure 3C) [57]. This arrangement closely resembled that observed in tubular SVPs [58] and infectious Dane particles [58,59], but differed significantly from recombinant SVPs [55]. Central sections of the cryo-EM maps identified a ~50 Å-thick lipid bilayer, with densities corresponding to the transmembrane domains of HBsAg penetrating the bilayer and forming the surface protrusions. Interestingly, the density of the lipid bilayer was comparable to that of proteins, which is inconsistent with the expected lower electron scattering of fluid lipids, leaving the membrane organization inconclusive. Asymmetry in the cryo-EM structure revealed variation in the size and spacing of the spikes across the particle surface. The study attributed this variation to differences in the presence and arrangement of S-HBs, M-HBs, and L-HBs proteins, resulting in a more divergent organization of HBsAg in tubular SVPs and Dane particles than spherical SVPs [57].

### 5.1. Molecular Architecture of WHV and HBV SVPs Resolved by Cryo-EM

**Figure 4:**
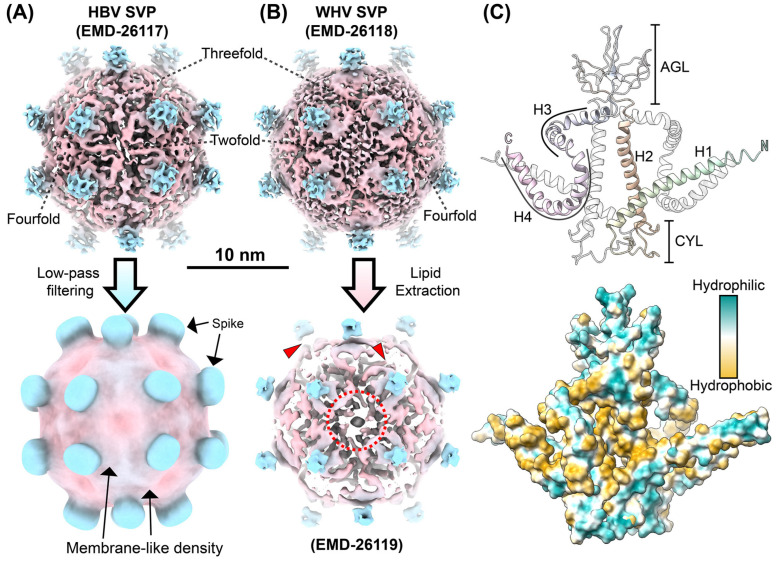
Structural features and lipid organization of HBV and WHV subviral particles analyzed by cryo-EM. (**A**,**B**) Sub-nanometer-resolution cryo-EM reconstructions of HBV (**A**) and WHV (**B**) SVPs reveal 24 protruding spikes arranged symmetrically [14]. Each spike comprises a dimer of small surface antigens (S-HBs or S-WHs), where each dimer contains two chemically identical monomers adopting slightly different conformations. These conformational differences primarily arise from the flexibility of the loop regions connecting the helical domains. When the 3D cryo-EM maps are low-pass filtered, detailed features of the protein and lipid densities smear together, giving the false appearance of a continuous lipid bilayer at the particle surface. Lipid extraction with 0.1% NP-40 overnight removes lipid patches (red dashed circle), destabilizing helices and exposing hydrophobic residues to the aqueous environment, resulting in the disappearance of associated density (red arrowheads). (**C**) The atomic model (PDB Code: 7TUK) is generated by integrating AlphaFold2-predicted structures with cryo-EM density maps, incorporating known protein–protein interactions. The upper panel shows the protein backbone of the S-HBs dimer with annotated structural features, including helices (H1–H4), the antigenic loop (AGL), and the cytosolic loop (CYL). One monomer is shown in color, while the other is in white. The dimer is stabilized by hydrophobic interactions between H1/H4 and H2/H2. Additionally, disulfide bonds formed at the AGL and CYL also contribute to intermolecular interaction. Due to unresolved cryo-EM density, flexible loops (AGL and CYL) could not be modeled correctly. The lower panel illustrates the hydrophobicity surface, highlighting hydrophobic regions sequestered within lipid patches. Structural comparisons indicate that HBV and WHV SVPs adopt distinct S-HBs orientations and incorporate lipids differently. This results in an approximately 70° rotation between dimers within WHV and HBV SVPs, leading to distinct dimer–dimer interactions at the symmetrical interfaces. These variations stabilize exposed hydrophobic residues, thereby contributing to overall particle integrity.

We recently obtained the first sub-nanometer-resolution structures of SVPs from HBV, isolated from the serum of a chronically infected patient, and woodchuck hepatitis virus (WHV), purified from a woodchuck infected with WHV (strain 7). These structures were resolved to 6.3 Å (Figure 4A, upper panel) and 6.8 Å (Figure 4B, upper panel), respectively, using cryo-EM in combination with AlphaFold2 predictions [14]. Both particles exhibit rhombicuboctahedral symmetry, a polyhedral structure characterized by twofold, threefold, and fourfold rotational axes. Approximately 23 nm in diameter, these particles feature 24 protruding spikes, each representing a conformationally heterogeneous dimer of S-HBs proteins. Each S-HBs monomer consists of four helical domains (H1–H4). The H1 and H2 helices, which are the longest and straightest, are separated by a cytosolic loop (CYL) containing a zinc finger motif [61]. Following the H2 helix is the AGL (Figure 2B), which consists of eight cysteine residues that form crosslinks with the neighboring AGL during dimerization. Together, the two AGLs form the “a” determinant, a critical immunogenic region, and the primary target of neutralizing antibodies [62,63]. The amphipathic H3 helix continues beyond the AGL, lying flat on the particle surface in a V-shaped conformation. Its hydrophilic side faces outward, while its hydrophobic residues form a structural framework interacting with the neighboring H3 helix and the C-terminal end of the H4 helix. The H4 helix adopts a U-shaped conformation, with its N-terminal end curving inward and its C-terminal end looping back toward the particle surface. The central residues of this U-shaped helix establish a hydrophobic core that contributes to the stability of the SVP structure, particularly around the twofold axes.

The protein architecture is further stabilized by the natural arrangement of hydrophobic and hydrophilic residues. Hydrophilic regions are exposed on the inner and outer surfaces of the particle, while hydrophobic residues are sequestered within the core (Figure 4C, lower panel). This organization ensures stability in aqueous environments, with the hydrophilic exterior shielding the hydrophobic interior. Unlike conventional lipid bilayers, the lipid organization in these SVPs consists of discrete lipid patches embedded within the densely packed protein surface. These lipid patches safeguard hydrophobic protein residues from the aqueous environment, enhancing particle stability through hydrophobic and electrostatic interactions. Cryo-EM density maps revealed a disorganized outer lipid layer resembling a mesh interspersed with protein density rather than a cohesive bilayer.

Interactions between lipid patches and protein regions near the rotational axes play a key role in particle stability. Within the particle, tightly packed residues in the H1–H4 helical regions form a hydrophobic core environment that maintains structural integrity, even in the absence of a traditional lipid bilayer. Lipid extraction experiments confirmed the importance of lipid patches as treatment with NP-40 disrupted the HBV SVPs, while WHV SVPs remained more resistant due to higher or tighter association with lipid content. Cryo-EM 3D reconstructions of NP-40-treated SVPs revealed the loss of lipid moieties and partial unwinding of helices in lipid-enriched regions, consistent with earlier observations (Figure 4B, lower panel) [44].

### 5.2. Structural Dynamics and Lipid Variability in Recombinant HBV SVPs

The structure of recombinant small spherical HBV SVPs, purified from FreeStyle 293F cells, was resolved at 3.6 Å, revealing pseudo-octahedral symmetry with arrangements of 3 × 2-mer hexamers around a threefold symmetry axis [61]. Cryo-EM analysis initially achieved an asymmetrical reconstruction at 4.7 Å resolution. Imposing C3 symmetry improved the resolution slightly to 4.24 Å, highlighting the conformational heterogeneity of the SVP assembly. Further refinement using subtracted sub-particles along different symmetrical facets enhanced the resolution to 3.1 Å. This strategy demonstrated that interactions between these facets were either misaligned with the imposed symmetry or inherently flexible. Consequently, processing data at the sub-particle level reduced conformational variability and improved the final structure. Altogether, these results suggest that although SVPs exhibit pseudo-octahedral symmetry, the subunits within the particles remain dynamic, likely due to variations in lipid composition and lipid–protein interactions.

Simultaneously, Wang et al. resolved SVP structures purified from CHO and HEK293 cell lines using cryo-EM. Their analysis revealed that the SVP organization establishes twofold and fourfold symmetry axes, forming a stable structure that deviates from perfect octahedral symmetry. Further analysis identified two quasi-symmetries in recombinant SVPs, D2 and D4-like symmetries [64]. D2 particles contained 80 S-HBs proteins (40 spikes), while D4 particles included 96 S-HBs proteins (48 spikes), forming a denser arrangement. This structural divergence highlighted the ability of HBsAg to adopt multiple configurations, reflecting variations in lipid–protein interactions and assembly conditions. Sub-particle reconstruction further refined the structure of S-HBs to 3.67 Å resolution. Unlike native HBV and WHV SVPs, which display patchy lipid organization [14], recombinant SVPs exhibited a distinct lipid bilayer that accommodates the transmembrane regions of S-HBs proteins. This bilayer contributes to the stability and formation of surface protrusions. The study proposed that the complete lipid bilayer observed in recombinant SVPs is influenced by the production system and assembly mechanisms, distinguishing it from the patchy lipid organization observed in native SVPs.

Closer examination of the lipid bilayer density in the Wang et al. study [64], however, revealed ambiguities that require further clarification. First, the continuous lipid density emerges at intermediate resolutions (see Figure S9 in [64]), where smeared densities pose difficulty in distinguishing lipids from protein signals. In a true lipid bilayer, the lipid density should appear weaker than the protein density due to its fluidity, lack of ordered structure, and lower atomic weights of lipids compared to proteins. For comparison, the lipid bilayer of the dengue virus, originating from the ER membrane, exhibits weaker density than its envelope proteins in both 2D classes and 3D reconstructions [65]. Second, segmented lipid signals in medium-resolution maps revealed large lipid masses at the outer layer, some even larger than the protruding spikes (see Figure S6 in [64], purple color). These densities, however, disappeared in high-resolution reconstructions, raising questions about their validity. Additionally, the resolution of D2 and D4 particles was limited to 6.59 Å and 8.47 Å, respectively, suggesting significant flexibility in S-HBs within these SVPs.

**Figure 5:**
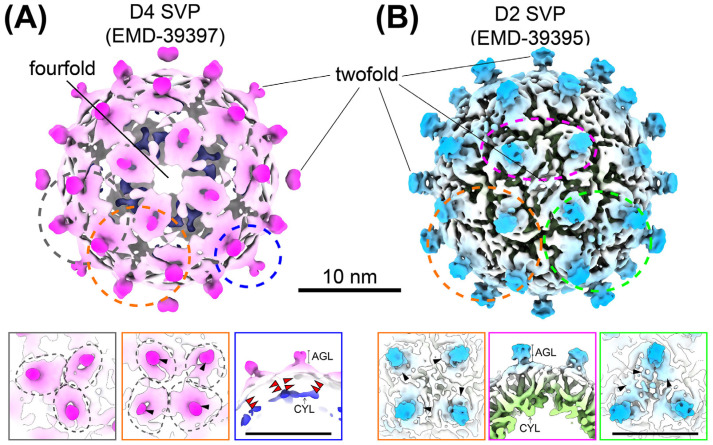
High-resolution structural analysis of recombinant HBV SVPs with D4 and D2 symmetries. (**A**) The recombinant HBV SVP with D4 symmetry, resolved to 8.5 Å, exhibits noticeable structural heterogeneity [64]. Protruding spikes vary in shape and density, with some appearing smaller in mass. Around the pseudo-threefold axis (black dashed circle), three spikes show distinct conformations of helices lying parallel to the particle surface: one forms a V-shape, while the others adopt rhomboid-like shapes. At the pseudo-twofold or pseudo-fourfold positions (orange circle), one spike consistently shows reduced density (black arrowheads). Transmembrane helix densities are absent (red arrowheads), while helices lying parallel to the particle surface remain visible (blue box). These findings suggest that spikes may not align perfectly with the imposed D4 symmetry (4-2-2 operations), potentially contributing to uneven density during averaging. (**B**) The recombinant HBV SVP with D2 symmetry (2-2-2 operations), resolved to 6.6 Å, shows improved structural quality, with S-HBs subunits closely resembling published structures [14,61]. However, density discrepancies persist (black arrowheads), possibly reflecting uneven lipid moieties bound to hydrophobic regions. In both D4 and D2 structures, no continuous lipid membrane density is observed at the reported resolution. At lower contour levels, smeared density at the outer lipid layer suggests a disordered lipid arrangement. The lower panels highlight corresponding regions of interest in D4 and D2 SVPs, illustrating spike variability, helical density loss, and lipid interactions. Scale bar, 10 nm.

For the SVP with D4 symmetry, an early-stage analysis of 3D classes with C1 symmetry showed incomplete and asymmetrical particles, where spikes were clustered on one side while the opposite surface appeared smooth (see Figure S5 in [64]). After particle selection, variations in spike density and distribution across particles persisted, indicating non-uniform spike positioning. Imposing D4 symmetry revealed distinct spike conformations; some were aligned with V-shaped helices parallel to the particle surface, while others adopted rhomboid-like shapes (Figure 5A). Notably, transmembrane helical densities were absent, while surface helices remained visible (Figure 5A, lower panel, blue box). These discrepancies indicate that the application of incorrect symmetry during data processing may have introduced artifacts. Conversely, D2 SVPs displayed more intact and symmetrical particles, though minor density variations persisted in the spikes. D2 symmetry included three perpendicular twofold axes, with identical S-HBs subunits at these positions (Figure 5B). However, the spike densities among these axes were inconsistent, contradicting the proposed regular threefold conformation of the spikes (Figure 5B, lower panel, green box). One dimer displayed smeared or porous density along parallel helices, potentially due to improper symmetry averaging or lipid-induced variability.

**Figure 6:**
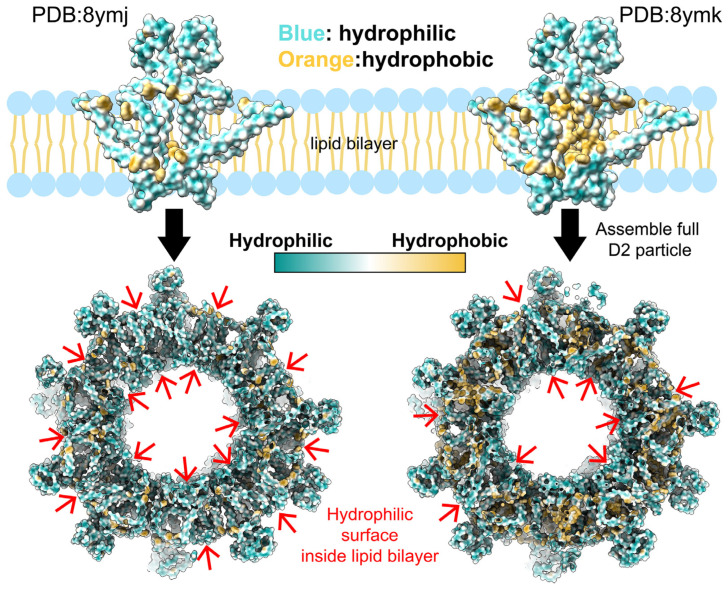
Surface hydrophobicity analysis of recombinant S-HBs raises questions about lipid bilayer compatibility. Two atomic models of recombinant HBV S-HBs, derived from high-resolution cryo-EM structures (PDB: 8ymj and 8ymk) [64], challenge the traditional concept of a lipid bilayer. The top panels depict the hydrophobicity surface of S-HBs dimers (blue: hydrophilic; orange: hydrophobic). In the D2 cryo-EM structure at 6.6 Å resolution (**left**, PDB: 8ymj), the protein surface is predominantly hydrophilic, with minimal hydrophobic regions, raising doubts about its ability to embed into a lipid bilayer. A more refined model at 3.67 Å resolution (**right**, PDB: 8ymk), derived from using sub-particle data processing, resolves residue sidechains but still includes several hydrophilic transmembrane regions. The lower panels show assembled D2 particles, highlighting multiple hydrophilic transmembrane regions (red arrows). The helices exhibit the following distinct hydrophobic properties: the V-shaped helices are amphipathic, with both hydrophobic and hydrophilic surfaces, while the U-shaped helices are predominantly hydrophobic, as reported in the literature [66,67]. Despite these features, the overall hydrophilic surface of the assembled structures poses questions about how these proteins integrate into a lipid bilayer. While hydrophilic and electrostatic interactions support structural integrity, further investigation is needed to understand the lipid–protein interactions involved in HBV SVP assembly and stabilization.

Furthermore, atomic models derived from these cryo-EM studies revealed highly hydrophilic protein surfaces (Figure 6). The V-shaped helices were identified as amphipathic [66], while the U-shaped helices were primarily hydrophobic [67], consistent with the prior literature. However, the overall hydrophilic nature of the assembled S-HBs raises questions about how such proteins could anchor into a lipid bilayer. Although hydrophilic and electrostatic interactions may contribute to the structural integrity of SVPs, these findings highlight the need for further exploration of the lipid–protein interactions that govern SVP assembly and stabilization.

### 5.3. Disassembly and Reassembly of HBV SVPs

The structural stability of SVPs depends on both hydrophobic and covalent interactions. Earlier attempts to extract lipids from SVPs using detergents showed that the particles retained their antigenicity under mild treatment [37,43,44]. Treating WHV SVPs with 1% NP-40 overnight leaves some intact particles, indicating their resistance to complete disassembly [14]. However, more detailed studies demonstrated that successful SVP disassembly requires both a reducing agent to disrupt disulfide bonds and a detergent to disrupt hydrophobic interactions. Treatments with urea, salt, or heat alone were insufficient, demonstrating the critical role of hydrophobic interactions in maintaining SVP integrity [68].

Disassembly was achieved by combining DTT, which reduces intermolecular disulfide bonds, with non-ionic detergents such as Triton X-100 or a mixture of Triton X-100 and ionic detergents like SDS. This treatment fragmented SVPs into smaller subunits. TEM imaging of disassembled particles revealed a significant loss of structural integrity, with protein components migrating to the top of sucrose density gradients. However, DTT alone was ineffective without detergents, highlighting the interplay between covalent and hydrophobic interactions in stabilizing SVPs.

Reassembly of SVPs was accomplished by removing detergents from disassembled S-HBs subunits [68]. The resulting particles displayed a broader size distribution under TEM, ranging from 12 to 68 nm in diameter. These reassembled particles were larger and morphologically distinct compared to native SVPs, suggesting a partial loss of structural fidelity during reassembly. Although the reassembled particles regained structural stability, defined here as the ability to assemble into particle-like structures, their reactivity with polyclonal antibodies targeting native conformational epitopes was reduced. This reduction suggests that critical epitopes were rearranged during reassembly, compromising the functional integrity of the particles.

These findings highlight the importance of hydrophobic interactions and lipid moieties in maintaining the structural and functional integrity of SVPs.

## 6. Conclusions Remarks

Despite being classified as an enveloped DNA virus, HBV’s mechanism for membrane fusion during host cell entry remains elusive, leaving a significant gap in our understanding of its molecular virology. HBV SVPs, which share the same envelope proteins and lipids as the virus and are derived from the endoplasmic reticulum membrane, present a promising avenue for addressing this challenge. Investigating the structural nature of the SVP membrane is crucial to unraveling the complexities of HBV infection. This question has been the subject of scientific discussion for over 50 years, yet definitive answers remain out of reach.

Advancing our understanding of SVP membrane architecture is not merely an academic pursuit; it has far-reaching implications. By uncovering the intricate details of lipid organization, symmetry, and protein dynamics, we can bridge the gaps in HBV molecular virology and refine therapeutic and preventive strategies. SVPs are a remarkable model for structural adaptation in viral assembly, exhibiting a unique combination of lipid–protein interactions and symmetry that sets them apart from other viral and lipoprotein complexes.

Recent advances in cryo-EM and molecular modeling have illuminated the complexity of SVP architecture, particularly the interplay between hydrophobic and covalent interactions that govern their stability and function. However, many challenges remain. Resolving the organization of the lipid membrane and the conformational dynamics within these particles is a critical frontier in structural virology.

The study of SVP disassembly and reassembly highlights the critical role of lipid–protein interactions in maintaining antigenicity and structural fidelity. These findings deepen our fundamental understanding while inspiring new approaches to engineering SVPs as vaccine platforms. Optimizing lipid and protein configurations and their interplay remains a significant challenge. Addressing these complexities could drive innovations that enhance immunogenicity and expand the applications of SVPs in combating HBV and other related diseases. Future research should integrate high-resolution structural techniques, such as advanced cryo-EM and single-molecule approaches, with biochemical and biophysical analyses. Collaborative efforts across disciplines will be crucial for unraveling the mysteries of SVP assembly, stability, and functionality. By resolving these unanswered questions, we can unlock new opportunities for breakthroughs in HBV treatment, prevention, and, ultimately, eradication.

## Data Availability

This review does not include newly generated data. However, the original structural data referenced in this study are summarized below. All cryo-EM 3D maps can be accessed from the Electron Microscopy Data Bank (https://www.ebi.ac.uk/emdb/, accessed on 24 September 2024): EMD-1158 and EMD-1159 (Gilbert et al. [55]), EMD-6954 (Cao et al. [57]), EMD-26117, EMD-26118, and EMD-26119 (Liu et al. [14]), and EMD-39395 and EMD-39397 (Wang et al. [64]). The corresponding atomic models are available from the Protein Data Bank (https://www.rcsb.org/, accessed on 24 September 2024): PDB-7TUK (Liu et al. [14]) and PDB-8YMJ and PDB-8YMK (Wang et al. [64]).
